# Effects of Leucosporidium‐derived ice‐binding protein (LeIBP) on bull semen cryopreservation

**DOI:** 10.1002/vms3.269

**Published:** 2020-04-22

**Authors:** Hoon Jang, Hyo J. Kwon, Wu S. Sun, Seongsoo Hwang, In S. Hwang, Sungwoo Kim, Jun H. Lee, Sung G. Lee, Jeong W. Lee

**Affiliations:** ^1^ Biotherapeutics Translational Research Center Korea Research Institute of Bioscience and Biotechnology Daejeon Korea; ^2^ Animal Biotechnology Division National Institute of Animal Science Wanju Korea; ^3^ Animal Genetic Resources Station National Institute of Animal Science Namwon Korea; ^4^ Department of Polar Bioconvergence Research Division of life Science Korea Polar Research Institute Incheon Korea

**Keywords:** Antioxidant activity, Bull sperm, Cryopreservation, LeIBP

## Abstract

We examined the effect of ice‐binding protein derived from *Leucosporidium* (LeIBP) on the cryopreservation of bull semen and compared it with that derived from previously reported Antifreeze Protein III (AFPIII). Six concentrations of LeIBP (10^–1^ ~ 10^4^ μg/ml) and AFPIII (10^–1^ ~ 10^4^ μg/ml) were added to the bull semen extender, respectively. Sperm kinematic parameters were measured to examine sperm toxicity and cryopreserved sperm quality. Measures of antioxidant activity such as superoxide dismutase (SOD), reduced glutathione/oxidative glutathione (GSH/GSSG), and total antioxidant capacity (TAC) were analysed to identify the effect of LeIBP on sperm quality. In addition, sperm viability was analysed using a flow cytometer and fluorescence microscope by SYBR14/PI staining. The results showed that the LeIBP groups (0.1, 1 and 10 μg/ml) were less toxic, and the quality of the sperm were dramatically improved in the extenders containing 0.1 μg/ml LeIBP among concentrations of LeIBP and AFPIII. The SOD activity of LeIBP was greater than that of AFPIII and control. In addition, sperm viability was enhanced in the LeIBP‐treated group. In summary, LeIBP is a useful cryoprotective adjuvant for bull sperm cryopreservation, and the most efficient concentration of LeIBP is 0.1 μg/ml.

## Graphical abstract

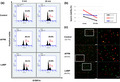

## 1 INTRODUCTION

Semen cryopreservation is a major part of artificial insemination, and it is the most broadly applied technique to preserve the genetic traits of cattle. For decades, the technology of efficient sperm preservation has been developed, but much of the sperm is internally damaged or immotile during freezing and thawing, which results in low fertilization rates (Layek, Mohanty, Kumaresan, & Parks, 2016). Several studies have demonstrated that oxidative stress is a major cause of sperm damage during the freezing and thawing process, and many researchers were performing studies on the effect of antifreeze protein on cryopreservation of sperm (Kumar et al., 2018; Qadeer et al., 2014; Sharma & Agarwal, 1996; Tasdemir et al., 2013; Wagner, Cheng, & Ko, 2018; Zheng, Zhang, Liu, Li, & Jiang, 2017). A recent study reported that LeIBP enhanced the quality of oocyte and embryo development, and decreased the ROS in vitrification of mouse oocyte (Lee, Lee, et al., 2015). Therefore, further study of LeIBP on bull sperm cryopreservation is also needed.

Reactive oxygen species (ROS) are a main cause of sperm damage during the process of freezing and thawing (Baumber, Ball, Linfor, & Meyers, 2003) and triggers oxidative stress (Sariozkan, Bucak, Tuncer, Ulutas, & Bilgen, 2009), which induces malfunction of the plasma membrane, reduction in mitochondrial membrane potential and DNA damage (Aitken & Krausz, 2001). Several studies have suggested that an anti‐oxidative agent successfully reduced ROS during sperm cryopreservation (Bucak et al., 2010; Michael et al., 2009). Recent studies showed that anti‐freezing protein (AFP, derived from fish) reduces ROS during the cryopreservation of oocytes and sperm (Jo, Jee, Lee, & Suh, 2011; Lee, Lee, et al., 2015).

Ice‐binding proteins (IBPs), a type of AFP, bind to crystalized ice and suppress its development and re‐crystallization. Ice crystallization causes critical damage to cellular membranes (Knight, DeVries, & Oolman, 1984; Raymond & Fritsen, 2001; Raymond & Knight, 2003). Because researchers have suggested that IBPs have potential use in academia and industries (Lee, Lee, Kim, & Hong, 2018), IBPs in many polar organisms, such as bacteria (Garnham et al., 2008), fungi (Xiao et al., 2010), plants (Middleton, Brown, Davies, & Walker, 2009) insects (Leinala et al., 2002) and fish (Chen & Jia, 1999), have been characterized. Recently*,* Lee et al. isolated extracellular IBP from arctic yeast *Leucosporidium* sp. AY30 (LeIBP) (Lee et al., 2010). The study demonstrated that LeIBP is different from other IBPs in terms of amino acid sequences but is similar to hyperactive AFPs in terms of its three‐dimensional structure (Lee, Park, et al., 2012; Park et al., 2012). LeIBP reduced the haemolysis of human red blood cells (Lee, Park, et al., 2012), decreased DNA double‐strand breaks in oocytes (Lee, Lee, et al., 2015) and protected ovarian tissue (Kong et al., 2018; Lee, Lee, et al., 2015) during cryopreservation. Based on the characteristics of LeIBP, we examined the protective effect of bull sperm freezing to enhance the success of the artificial insemination. We added LeIBP to the extenders and investigated the toxicity and protective effect in a dose‐dependent manner during semen cryopreservation. LeIBP was compared to AFPIII (0.1 μg/ml), which was reported to improve Nili‐Ravi sperm motility and plasma membrane integrity (Qadeer et al., 2014). In addition, we demonstrated that LeIBP plays an important role in the reduction of oxidized glutathione among several antioxidant effects, and improvement of sperm viability.

## 2 MATERIALS AND METHODS

### 2.1 Animal ethics

The protocol and procedures for the treatment of bulls were approved by Institutional Animal Care and Use Committee of the National Institute of Animal Science.

### 2.2 Study design

This study was designed as follows. We collaborated with a research team studying lineage preservation in traditional Korean cattle to compare LeIBP, a purified recombinant protein, with AFPIII protein, which has been reported to have a cryoprotective effect. Cryoprotective effects were analyzed based on kinetic and directionality by measuring kinematic parameters of sperm. Total antioxidant efficiency was measured to see the effect of oxidative stress reduction. In addition, SYBR14/ propidium iodide (PI) staining was performed to confirm the survival rate of sperm after freeze–thaw using imaging and cytometry.

### 2.3 Extender preparation

The extender was prepared using a solution consisting of 20% egg yolk and 20% Triladyl (Minitube, Germany) in distilled water. Recombinant LeIBP was provided by Dr. Kim (Lee et al., 2013; Lee, Park, et al., 2012). The normal extender was used as the control, and treatments of 0.1, 1, 10, 10^2^, 10^3^ and 10^4^ μg/ml LeIBP and AFPIII were added to the extender, respectively. In the extender including AFPIII (A/F PROTEIN, USA), 0.7% of phenoxyethanol (Sigma‐Aldrich, USA) was added to complete dissolution according to the manufacturer's suggested protocol.

### 2.4 Semen collection and evaluation of the toxic effect of LeIBP

Fresh semen was collected from five healthy Korean bulls aged 50–65 months using an artificial vagina for each experiment (at least three replicates). Sperm collection was carried out every two weeks, ejaculation was performed once. Ejaculated semen was transported to the research laboratory at 28 ℃, and the quality was immediately analysed using a computer‐associated sperm analysis (CASA) system (PROiSER, UK). The CASA system setup was basically referred to the manufacturer's manual and the measured sperm concentration was adjusted to less than 10^7^/ ml. We selected semen of good quality (˃ 90% forward progressive motility and concentrations of at least 2 × 10^8^ sperm/ml). After the evaluation of sperm quality, the fresh semen was divided into seven equal fractions to identify the toxic effect of LeIBP. The LeIBP was added at doses of 10^−1^,10^0^, 10^1^, 10^2^, 10^3^, and 10^4^ μg/ml to make experimental extenders. A control contained no LeIBP. The semen aliquots (1 × 10^7^ sperm/ml) were then incubated with the extenders for 24 hr at 17℃.

### 2.5 Semen Freeze and thaw, and analysis of kinematic parameters

The fresh semen (at least 2 × 10^8^ sperm/ml) was divided into four equal fractions (1 × 10^7^ sperm/ml) in a water bath at 3℃; one fraction was diluted with the extender for the control group and the other fractions were diluted with extenders including doses of LeIBP (0.1, 1, and 10 μg/ml). The semen samples were slowly cooled to 5 ℃ for 3 hr (−10℃/hr) in a water jacket. Next, it was loaded into 0.5 ml straws and then placed 4 cm above the surface of liquid nitrogen for 10 min according to the previous report (Santos, Sansinena, Zaritzky, & Chirife, 2013). Next, the straws were immersed directly into liquid nitrogen for storage. To thaw the semen sample, the straws were placed into a water bath at 37 ℃ for 50 s, and sperm quality was then analysed.

### 2.6 Detection of antioxidant enzymatic activities

Thawed semen samples were centrifuged 1,500g for 5 min at 25℃. The pellet was washed twice with Phosphate‐buffered saline (PBS) and re‐suspended with 500 μl of PBS including 1% Triton X‐100 for 20 min for the extraction of enzymes. After incubation, the samples were centrifuged for 30 min at 4,000g, and the supernatant was transferred to a new tube for the consequent procedures. A Total antioxidant capacity (TAC) assay kit (DoGen, Korea), EZ‐SOD assay kit (DoGen, Korea) and an EZ‐glutathione (GSH) assay kit (DoGen, Korea) were used to measure the antioxidant enzymatic activity according to the manufacturer's manual. Quantification relative to total protein was then performed using a DC Protein Assay Reagent system (BIO‐RAD, USA).

### 2.7 Staining of bull sperm for flow cytometry and microscopy

Thawed semen samples were centrifuged 1,500g for 10 min at 25 ℃. The pellet was washed twice and re‐suspended with 1 ml of PBS including 10% Bovine serum albumin (BSA). The sperm sample was incubated for 30 min in 20 μM of SYBR and 14 and 12 μM of PI at 30℃ in a dark incubator. Next, the sperm samples were left at room temperature for 20 min to measure the survival rate by time. To analyse sperm viability using an Accuri C6 flow cytometer (BD Bioscience, USA), the samples were diluted to 1 x 10^5^ sperm/ml with PBS including 10% BSA solution to reduce the sperm concentration. The number of sperm stained in green (FL1‐A) was divided by the total number of sperm (green, orange and red) to measure viability for each. To confirm the viability of sperm, each group of sperm samples was measured using IX83 fluorescence microscope (OLYMPUS, Japan).

### 2.8 Statistical analysis

For reduction of error, two straws in each treatment were thawed separately. Five different microscopic fields in each group were analysed during operation of the CASA system. At least three independent experiments were performed. All results were expressed as mean value ± *SEM*. Tukey's test was used as a post‐hoc test, and a one‐way analysis of variance (ANOVA) procedure was used to compare the mean value of the sperm kinematic parameters and enzymatic activity. The level of significance was set at *p* < .05.

## 3 RESULTS

### 3.1 Toxicity of the LeIBP on fresh sperm

To examine the sperm toxicity of LeIBP, doses of LeIBP (10^–1^,10^0^, 10^1^, 10^2^, 10^3^ and 10^4^ μg/ml) were added to bull semen extenders at 17 ℃ for 24 hr. After incubation, the sperm kinematic parameters were measured using a CASA system. To compare LeIBP with AFPIII, the same concentrations of AFPIII were also analysed. The results showed that sperm kinetics were significantly different from that of the control group in LeIBP (10 mg/ml), but no differences were observed in all concentrations of AFPIII groups (Table 1). As sperm linearity was significantly reduced at concentrations above 100 μg/ml LeIBP compared to the control group, we investigated the cryoprotective effects of concentrations below 10 μg/ml LeIBP (10, 1 and 0.1 μg/ml). Taken together, we conclude that high concentrations of LeIBP, unlike those of AFPIII, have abnormal effects on bull sperm quality.

**Table 1:** Toxic effect of LeIBP and AFPIII on the kinematic parameters of fresh sperm

	Control	LeIBP						AFPIII	
		0.1 μg/ml	1 μg/ml	10 μg/ml	100 μg/ml	1 mg/ml	10 mg/ml	0.1 μg/ml	10 mg/ml
LM (%)	97.51 ± 0.54^a^	97.70 ± 0.58^a^	97.47 ± 1.15**a**	97.47±1.15**a**	96.83 ± 1.70**a**	98.62 ± 0.37**a**	89.59 ± 1.37**b**	97.64 ± 1.24a	98.43 ± 0.37a
VCL (μm/s)	111.87 ± 0.84^a^	113.12 ± 1.20^a^	128.54 ± 2.70^b^	128.54 ± 2.70^ b^	114.07 ± 2.21^a^	127.04 ± 2.86^bd^	100.97 ± 3.68^a^	111.64 ± 1.15^a^	122.41 ± 3.82^a^
VSL (μm/s)	38.68 ± 0.15^a^	41.06 ± 0.22^ab^	43.25 ± 0.83^b^	43.25 ± 0.83^b^	43.04 ± 0.36^b^	36.25 ± 0.11^ac^	28.40 ± 1.13^d^	43.77 ± 2.07^b^	37.61 ± 0.85^a^
VAP (μm/s)	56.81 ± 0.45^a^	58.86 ± 0.95^ab^	66.12 ± 1.91	66.12 ± 1.91^bc^	60.83 ± 1.11^ab^	58.98 ± 0.83^abc^	45.63 ± 1.64^d^	59.30 ± 0.55^a^	59.46 ± 1.76^a^
LIN (%)	34.58 ± 0.15^a^	36.30 ± 0.19^ab^	33.65 ± 0.07^a^	33.65 ± 0.07^a^	37.76 ± 0.76^b^	28.57 ± 0.71^c^	28.12 ± 0.29^c^	39.23 ± 2.03^b^	30.76 ± 0.66^c^
STR (%)	68.09±0.52^ab^	69.78 ± 0.77^a^	65.45 ± 0.79^bce^	65.45 ± 0.79^bce^	70.79 ± 1.04^a^	61.50 ± 1.03^d^	62.21 ± 0.26^de^	73.76 ± 2.92^a^	63.31 ± 1.19^a^
BCF (Hz)	9.50 ± 0.11^a^	9.49 ± 0.29^a^	8.83 ± 0.16^a^	8.83 ± 0.16^a^	9.42 ± 0.20^a^	6.69 ± 0.38^b^	5.57 ± 0.23^c^	10.79 ± 0.30^a^	8.89 ± 0.11^a^
WOB (%)	50.78±0.30^a^	52.03±0.34^ab^	51.42 ± 0.51^ac^	51.42 ± 0.51^ac^	53.33 ± 0.53^bcd^	46.44 ± 0.42^e^	45.20 ± 0.38^e^	53.13 ± 0.72^a^	48.59 ± 0.18^a^
ALH (μm)	4.06 ± 0.02^ab^	3.95 ± 0.03^a^	4.50 ± 0.05	4.50 ± 0.05^ab^	4.03 ± 0.09^ab^	5.22 ± 0.08^c^	4.59 ± 0.28^b^	3.85 ± 0.03^a^	4.36 ± 0.11^b^

Note

Values within a row without a common superscript (a–e) indicate differences (*P˂ 0.05*). Values are represented as mean ± *SEM* of bull sperm in all the concentrations of LeIBP and AFPIII versus. control. All treatments were replicated three times. ALH, amplitude of lateral head displacement (μm); BCF, beat cross frequency (Hz); LIN, linearity (%);LM, percentage of linear motility (%);STR, straightness (%);VAP, velocity of the average path (μm/s); VCL, velocity of curvilinear (μm/s); VSL, velocity of straight‐line (μm/s); WOB, wobble (%).

### 3.2 Effect of LeIBP on sperm cryopreservation

To observe whether LeIBP has a protective effect on bull sperm cryopreservation, we performed semen freezing with extenders including three concentrations of LeIBP (0.1, 1 and 10 μg/ml). Because previous study reported the 0.1 μg/ml AFPIII has protective effect on bull sperm cryopreservation, the 0.1 μg/ml AFPIII was analyzed to compare with LeIBP. The results showed that major kinematic parameters as sperm motility, velocity (percentage of linear motility [LM], velocity of curvilinear [VCL]) significantly improved in 0.1 μg/ml LeIBP compared to the control (Table 2). In addition, 0.1 μg/ml LeIBP was more efficient than 0.1 μg/ml AFPIII in terms of LM, VCL, velocity of straight‐line (VSL) and amplitude of lateral head displacement (ALH) (Figure S1). Overall, our results indicate that 0.1 μg/ml LeIBP has a greater cryoprotective effect than 0.1 μg/ml AFPIII.

**Table 2:** Effects of LeIBP and AFPIII on kinematic parameters after freezing‐thawing sperm

	Control	LeIBP			AFPIII
		0.1 μg/ml	1 μg/ml	10 μg/ml	0.1 μg/ml
LM (%)	79.01 ± 1.23^a^	91.07 ± 0.66^c^	85.51 ± 1.61^b^	76.77 ± 1.36^a^	86.35 ± 2.80^b^
VCL (μm/s)	79.47 ± 2.28^a^	87.57 ± 1.78^b^	73.08 ± 1.60^c^	71.23 ± 0.41^c^	81.58 ± 4.02^a^
VSL (μm/s)	38.70 ± 1.14^ab^	34.71 ± 0.92^bc^	39.35 ± 0.89^a^	33.56 ± 1.03^c^	29.86 ± 0.80^d^
VAP (μm/s)	55.45 ± 1.65^ab^	49.73 ± 1.31^a^	46.77 ± 1.18^ab^	42.45 ± 0.34^b^	42.78 ± 1.72^a^
LIN (%)	47.11 ± 0.79^a^	40.01 ± 0.27^b^	53.86 ± 0.64^a^	47.12 ± 1.44^c^	37.07 ± 1.75^b^
STR (%)	73.30 ± 1.14^b^	67.11 ± 1.81^a^	84.16 ± 0.45^bc^	79.03 ± 1.91^bd^	66.42 ± 0.27^a^
BCF (Hz)	8.57 ± 0.10^a^	8.61 ± 0.34^b^	10.18 ± 0.16^a^	9.65 ± 0.41^a^	7.43 ± 0.69^b^
WOB (%)	61.96 ± 1.47^a^	57.01 ± 0.69^b^	64.00 ± 0.57^a^	59.61 ± 0.67^cb^	53.24 ± 1.20^b^
ALH (μm)	2.68 ± 0.10^a^	3.31 ± 0.02^b^	2.74 ± 0.10^a^	2.93 ± 0.05^ab^	2.82 ± 0.01^a^

Values within a row without a common superscript (a–d) indicate differences (*p ˂* *.05*). Values are represented as mean ± *SEM* of thawed bull sperm in the concentrations of LeIBP and AFPIII. All treatments were replicated three times. LM, percentage of linear motility (%); VCL, velocity of curvilinear (μm/s); VSL, velocity of straight‐line (μm/s); VAP, velocity of the average path (μm/s); LIN, linearity (%); STR, straightness (%); BCF, beat cross frequency (Hz); WOB, wobble (%); ALH, amplitude of lateral head displacement (μm)

### 3.3 Effect of LeIBP on the anti‐oxidative effect of bull sperm

Because various studies reported that the protective effect of AFPIII is relevant with increasing antioxidant effect, we conducted to determine whether the protective effect of LeIBP is also derived from stimulating antioxidant activity. Total antioxidant capacity (TAC), superoxide dismutase (SOD) activity and glutathione reduced/oxidized (GSH/GSSG) ratio were measured in the cryopreserved sperm using enzymatic analysis. The results showed that TAC was high in both 0.1 μg/ml of LeIBP and 0.1 μg/ml of AFPIII compared to the control group. SOD activity was significantly increased in all concentrations of LeIBP but not in the 0.1 μg/ml AFPIII. However, the GSH/GSSG ratio was significantly elevated in 0.1 μg/ml AFPIII but it was shown to increase in 10 μg/ml LeIBP only. Thus, the cryoprotective effect of LeIBP in sperm freezing is closely related to antioxidant activity.

### 3.4 Effect of LeIBP on sperm viability

**Figure 1:**
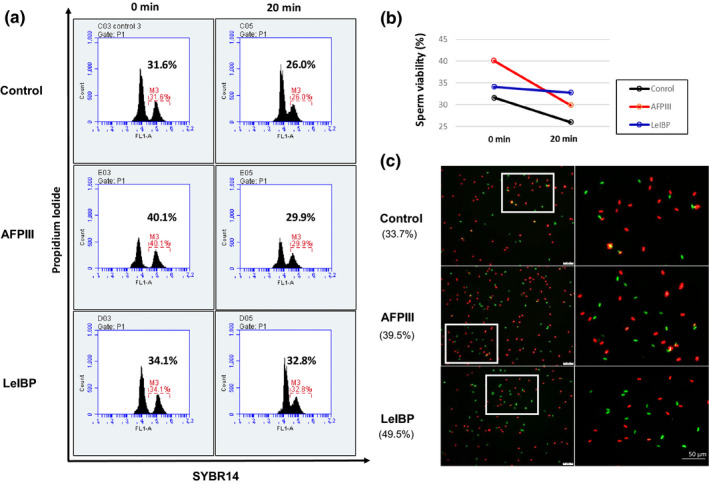
A comparative analysis of sperm viability in thawed sperm containing AFPIII (0.1 μg/ml) and LeIBP (0.1 μg/ml). Sperm viability was analysed by flow cytometry and fluorescence microscopy using SYBR14/PI staining. (a) Two peaks are shown by the intensity of FL1‐A (SYBR 14; green), and the intensity of the right peak, which is indicating sperm viability, was measured and compared. The intensity of FL1‐A was re‐measured after 20 min at room temperature. (b) The graph is added to ease comparison of the peak. (c) Green dots represent living sperm and red dots represent dead sperm. Sperm survival rate was calculated and indicated

To examine the effect of LeIBP in sperm viability, live‐dead analysis was performed. Thawed sperm was stained with SYBR14/PI, left at room temperature in a time‐dependent manner and viability was analysed using a flow cytometer and a fluorescence microscope. The flow cytometry analysis indicated that both AFPIII and LeIBP increased SYBR14‐positive sperm compared to the control group. In addition, analysis of the sperm survival rate over time confirmed that LeIBP affects sperm viability (Figure 1). Taken together, we found that LeIBP plays an important role in maintaining viability while increasing the survival rate of frozen‐thawed sperm.

## 4 DISCUSSION

Our findings indicate that LeIBP can be used as a successful protective agent in bull sperm cryopreservation. These results firstly suggest that the supplementation of LeIBP to the extender has a protective effect on sperm cryopreservation by including antioxidant activity. Previously, antifreeze and ice‐binding proteins have been studied to protect cryopreserved cells and tissues such as higher plants (Atici & Nalbantoglu, 2003), mammalian somatic cells (Kim, Shim, Lee, Kang, & Hur, 2015), oocytes (Jo et al., 2011) and sperm (Layek et al., 2016; Tasdemir et al., 2013). Researchers have been using various methods to study more effective cryoprotectants and their mechanisms in various cells. In sperm, the measuring kinematic parameters is the most important in identifying the quality of sperm after freeze–thaw procedures (Tasdemir et al., 2013). Therefore, the kinematic parameters were analysed to identify toxicity and protection effects after these procedures using the CASA system.

As the toxicity of LeIBP in sperm cryopreservation had not yet been reported, we firstly examined the toxicity of LeIBP in a dose‐dependent manner because the cryoprotectant should be not or less toxic by itself. We analysed the kinematic parameters and found that the LeIBP concentrations above 100 μg/ml show some differences among parameters when compared to the control and AFPIII groups (Table 1). However, several studies have reported that no toxicity was observed at concentration greater than 100 μg/ml LeIBP in somatic cells of ovarian follicles (Kong et al., 2018; Lee, Lee, et al., 2015), oocytes (Jo et al., 2011) or mammalian cells (Kim et al., 2015). Our results showed that the velocity of sperm such as VCL, VSL and VAP significantly increased at 1 μg/ml LeIBP, and sperm linearity dramatically decreased with concentrations above 1 mg/ml. In this regard, toxicity of LeIBP by concentration seems to be cell‐specific. Because sperm is more sensitive compared to other cells, we examined low concentrations of LeIBP (0.1, 1 and 10 μg/ml) in sperm cryopreservation. Interestingly, sperm toxicity was not observed at high concentrations of AFPIII (up to 10 mg/ml). Based on these facts, we suggest that toxicity tests on target cells will be necessary before LeIBP is applied.

The results of the LeIBP effect on sperm cryopreservation are interesting because sperm motility was quite enhanced compared to not only the control but also AFPIII. As AFPIII has been reported to have a cryoprotective effect not only in the spermatozoa of various animals (Nishijima et al., 2014; Qadeer et al., 2014) but also in many other cells (Antson et al., 2001; Atici & Nalbantoglu, 2003; Doucet et al., 2000; Jo et al., 2011; Lee, Lee, et al., 2015), the effect of LeIBP is more prominent. Most kinematic parameters were improved in 0.1 μg/ml LeIBP compared to control and AFPIII. In addition, the parameters began to decrease in the concentration of 10 μg/ml LeIBP or more, but there was no significant difference in 0.01 μg/ml LeIBP from the control group. Based on these facts, it can be inferred that the cryoprotective effect of LeIBP exists at an optimal concentration in each type of cell.

In a previous study, sperm was exposed to ROS during cryopreservation (Bilodeau, Blanchette, Cormier, & Sirard, 2002). The ROS is produced during the normal physiological process, and the accumulated metabolites of oxygen can induce lipid peroxidation (LPO) of the plasma membrane, which results in superoxide, hydroxyl radicals, and hydrogen peroxide during sperm physiological activity (Aitken & Baker, 2004; Witte et al., 2009). The mechanisms of glutathione peroxidase and SOD are cellular defense systems against LPO (Bilodeau et al., 2002), and the mechanisms of antioxidant activity can be determined by measuring each activity (Gadea et al., 2004). As recent studies discovered that antioxidants have a cryoprotective effect (Zheng et al., 2017), we examined the antioxidant effect of LeIBP. In this study, LeIBP stimulated the SOD activity, while AFPIII induced glutathione reductase activity (Table 3). Taken together, LeIBP and AFPIII have different antioxidant effects.

**Table 3:** Effect of LeIBP on the antioxidant enzymatic activities of sperm

	Control	LeIBP			AFPIII
		0.1 μg/ml	1 μg/ml	10 μg/ml	0.1 μg/ml
TAC (%)	100.01 ± 0.54^a^	105.94 ± 0.69^b^	107.81 ± 0.56^b^	102.7 ± 0.14^a^	109.22 ± 1.59^b^
SOD activity (%)	100.01 ± 1.36^a^	122.8 ± 2.49^b^	149.99 ± 22.95^b^	121.59 ± 5.27^b^	102.30 ± 6.21^a^
GSH/GSSG Ratio (%)	100 ± 6.28^a^	104.87 ± 2.05^a^	116.29 ± 4.72^a^	156.82 ± 16.61^b^	146.75 ± 7.27^b^

Note

Values within a row without a common superscript (a‐c) indicate differences (*p ˂ 0.05*).

Abbreviations: GSH, reduced glutathione; GSSG, oxidative glutathione; SOD, superoxide dismutase; TAC, total antioxidant capacity.

The ultimate goal of cryopreservation is to increase the efficiency of artificial insemination. Our results show that survival rates after freezing‐thawing of sperm treated with LeIBP and AFPIII are higher than those of the control group (Figure 1c). However, maintaining viability of sperm during in vitro fertilization (IVF) is also a critical factor in improving artificial insemination. Therefore, we examined sperm maintenance during times spent on artificial insemination, and found that sperm viability by LeIBP was superior to control and AFPIII. Based on these results, it is necessary to study the effect of LeIBP on the developmental rate of embryos after IVF, and to examine the synergic effect of sperm cryopreservation using a combination of AFPIII and LeIBP.

In conclusion, supplementation with LeIBP can allow for a greater cryoprotective effect and viability in bull sperm.

## CONFLICT OF INTEREST

None.

## AUTHOR CONTRIBUTION

**Hoon Jang:** Formal analysis; Writing‐original draft; Writing‐review & editing. **Hyo Jin Kwon:** Formal analysis; Visualization. **Wu Sheng Sun:** Validation; Writing‐review & editing. **Seongsoo HWANG:** Conceptualization; Funding acquisition. **In Sul Hwang:** Data curation; Resources. **Sung Woo Kim:** Conceptualization; Project administration. **Jun Hyuck Lee:** Investigation; Resources; Validation. **Sung Gu Lee:** Project administration; Resources. **Jeong‐Woong Lee:** Conceptualization; Project administration. 

## Supporting information

Figure S1

## Data Availability

Data sharing is not applicable to this article.
