# Supplementary material for: Effects of Leucosporidium‐derived ice‐binding protein (LeIBP) on bull semen cryopreservation
Source: Vet Med Sci. 2020 Apr 22;6(3):447–53. doi: 10.1002/vms3.269 (PMC7397894; doi:10.1002/vms3.269)
Supplement: Supplementary file 1 — Figure S1 [file VMS3-6-447-s001.docx]

**sFig. 1**. Comparison of sperm quality between AFP3 (0.1 μg/ml) and LeIBP (0.1 μg/ml) after freeze-thaw procedures. Anti-Freeze Protein III (AFP III) and *Leucosporidium*-derived ice-binding protein (LeIBP) were added to semen extenders, and cryopreservation was performed. After thawing, the kinematic parameters were measured using the CASA system. The experiments were repeated at least three times, and representative graphs are shown. **P ˂ 0.05, ** P ˂ 0.01.* Data are represented as mean ± SEM.

VAP (μm/s)

VSL (μm/s)

98.82±0.75^a^

128.32±1.69^a^

44.73±1.02^a^

68.14±0.94^a^

34.85±0.55^ac^

65.62±0.75^a^

8.99±0.10^a^

53.11±0.49^a^

4.44±0.11^a^

101.37±2.82^b^

42.10±1.22^a^

55.81±1.14^b^

41.61±1.81^b^

75.45±2.12^b^

10.58±0.15^b^

55.11±1.02^a^

3.55±0.06^b^

96.13±1.18^ab^

112.88±2.04^b^

44.31±0.53^a^

61.55±0.20^bc^

39.30±1.03^ab^

72.00±1.05^b^

9.54±0.34^bc^

54.56±0.93^ad^

3.92±0.05^b^

Control

1 μg/ml

10 μg/ml

100 μg/ml

1 mg/ml

AFP Ш

98.74±0.27^a^

131.32±2.29^a^

40.70±1.50^a^

65.00±1.51^a^

31.00±1.14^c^

62.57±0.96^ac^

8.09±0.33^a^

49.51±1.10^c^

4.67±0.20^a^

113.06±3.75^bc^

45.64±1.00^a^

61.69±1.47^bc^

40.46±1.45^b^

74.06±1.86^b^

10.51±0.25^b^

54.61±0.69^ad^

3.84±0.11^b^

95.53±0.77^b^

97.97±0.72^ab^

**sTable 1**. Sperm toxicity test of ATFIII

LM (%)

VCL (μm/s)

LIN (%)

STR (%)

BCF (Hz)

WOB (%)

ALH (μm)
